# Bioinformatics Analysis of Evolution and Human Disease Related Transposable Element-Derived microRNAs

**DOI:** 10.3390/life10060095

**Published:** 2020-06-25

**Authors:** Hee-Eun Lee, Jae-Won Huh, Heui-Soo Kim

**Affiliations:** 1 National Primate Research Center, Korea Research Institute of Bioscience and Biotechnology, Cheongju 28116, Korea; helee@kribb.re.kr (H.-E.L.); huhjw@kribb.re.kr (J.-W.H.); 2 Department of Functional Genomics, KRIBB School of Bioscience, Korea University of Science and Technology (UST), Daejeon 34113, Korea; 3 Department of Biological Sciences, College of Natural Sciences, Pusan National University, Busan 46241, Korea; 4 Institute of Systems Biology, Pusan National University, Busan 46241, Korea

**Keywords:** evolution, human disease, microRNA, transposable elements, transposable element derived microRNA

## Abstract

Transposable element (TE) has the ability to insert into certain parts of the genome, and due to this event, it is possible for TEs to generate new factors and one of these factors are microRNAs (miRNA). miRNAs are non-coding RNAs made up of 19 to 24 nucleotides and numerous miRNAs are derived from TE. In this study, to support general knowledge on TE and miRNAs derived from TE, several bioinformatics tools and databases were used to analyze miRNAs derived from TE in two aspects: evolution and human disease. The distribution of TEs in diverse species presents that almost half of the genome is covered with TE in mammalians and less than a half in other vertebrates and invertebrates. Based on selected evolution-related miRNAs studies, a total of 51 miRNAs derived from TE were found and analyzed. For the human disease-related miRNAs, total of 34 miRNAs derived from TE were organized from the previous studies. In summary, abundant miRNAs derived from TE are found, however, the function of miRNAs derived from TE is not informed either. Therefore, this study provides theoretical understanding of miRNAs derived from TE by using various bioinformatics tools.

## 1. Introduction

In 1986, transposable elements (TEs) were found from *Drosophila melanogaster* that have the ability to adjust their position and replicate in host genome that categorizes into two large classes with different mechanisms [1]. TEs are abundant in the genome of eukaryotes, which is about approximately from 3% up to 85% in the genome of plants and 50% in mammalian’s genome. The discovery on specific function of TE is still ongoing assignments for researchers, however, numerous studies revealed that TE is involved in epigenetics [2,3,4], evolution [5,6,7,8,9], and disease [10,11,12]. An interesting fact about class I TE called retrotransposons and evolution is that superfamily of retrotransposon long interspersed nuclear element 1 (LINE1 or L1), short interspersed nuclear element (SINE)-variable number of tandem repeat (VNTR)-*Alu* (SVA), and human endogenous retrovirus K (HERV-K) are active TEs in recent human evolution [13,14,15,16,17]. As mentioned earlier, not only in evolution, TE has very close relation with human cancer and disease [18,19,20,21,22,23,24,25,26,27,28,29]. For instance, L1 encodes RNA pol II promoter and this L1 promoter is hypomethylated in tumors of lung, colon, and several types of cancers [19]. TEs merge into the host genome, and this event has the possibility to provide and create new sequences to gene expression regulators such as microRNAs (miRNA) [4,30,31,32,33,34,35,36,37,38] and transcription factors (TF) [39,40,41].

In 1993, miRNA was first found in *Caenorhabditis elegans* (*C. elegans*) and it is comprised with 19 to 24 nucleotides of non-coding RNAs which is associated with gene regulations by targeting mRNAs for cleavage or translational inhibition [42,43,44,45]. The essential function of miRNA is correlated with oncogenesis, immunity, developments, and cell differentiations. Generally, 3′ untranslated region (UTR) is the miRNA binding sites of target mRNA. miRNA recognizes the complementary binding sites of target gene for its seed regions which is approximately 6 nucleotides long in miRNA. Previously, numerous studies have provided the evidence of identification on miRNA derived from TE [30,31,33,34,36] and some of the miRNAs derived from TE had a strong correlation with human disease [46,47,48,49,50,51,52] as well as evolution [53,54,55,56,57]. Considering the many substantial aspects of TE, miRNAs derived from TE mimic the functions of TE.

Bioinformatics tools are useful for the initial steps before starting the experiments, which is what understanding the primary information on what the study will be about. In the case of TE, analyzing the sequences and predicting structure of TE is important considering the function of TE. A TE-based database called ‘Repeatmasker’ provides the proportion of each type of TEs among diverse species [15]. Additionally, there are more of TE related bioinformatics tools and databases that determines which TE has merged into the target sequences, however, TE-based databases and programs are still limited [58,59,60,61]. In contrast with TE, numerous miRNA-based databases provide basic information about miRNA, miRNA related cancer, target genes, TFs, and so on [60,62,63,64,65,66,67].

In this study, evolution as well as human disease-related miRNAs derived from TE were examined through published research papers. Those determined miRNAs derived from TE were analyzed by using several bioinformatics tools to provide fundamental information of miRNAs derived from TE.

## 2. Bioinformatics Analysis of Transposable Elements

The distribution of TEs in various species (human, chimpanzee, gorilla, orangutan, gibbon, macaque, rhesus, marmoset, mouse, horse, cow, cat, dog, chicken, zebrafish, and *C. elegans*) were verified by RepeatMasker Genomic Datasets [15]. The species that are examined for whole genome sequencing are listed in both UCSC genome browser and RepeatMasker [15,60]. Table 1 shows the percentage of which TE is or not include in the genome. After the name of the superfamily element such as SINE, LINE, and long terminal repeats (LTR), the specific name of the element is given. The ‘other’ after the superfamily elements are representing the unspecified elements. The primates, including humans and the other seven of the species, are reasonably chosen to present the percentage of each TEs for evolutionary aspects.

Chicken had the lowest percentage of TEs in the genome (9.3%), and orangutan had the highest percentage of TEs in the genome (48.5%). From primates to mammalians (human, chimpanzee, gorilla, orangutan, gibbon, macaque, rhesus, marmoset, mouse, horse, cow, cat, and dog), variation of TE is well spread out in each species genome, excluding SVA element. SVA element was exclusive in humans. *C. elegans* is another species with the lowest percentage of TE in the genome (9%) and few of the TEs were included (LINE-CR1, LTE-other, DNA-TcMar, DNA-hAT, and DNA-other). Zebrafish is representing species of fish in this table and zebrafish contains the highest percentage of DNA transposons-other (19.6%).

## 3. Selection of Microrna Related Papers and Bioinformatic Analyses of Transposable Element-Derived microRNAs

**Figure 1:**
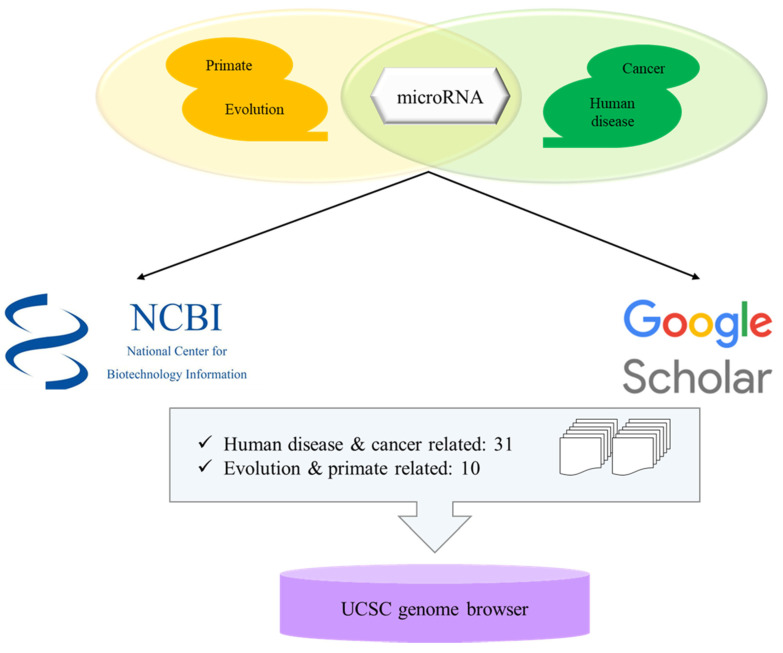
Schematic step of analyzing microRNAs derived from transposable elements.

miRNAs related with keywords of ‘evolution and primates’ and ‘human disease and cancer’ were searched from National Center for Biotechnology Information (NCBI)-PubMed database [68] and google scholar [69] (Figure 1). Each paper contained numerous miRNAs and the information of miRNAs were examined from miRbase v22.1 (http://www.mirbase.org) [66]. Then each miRNA was localized in human genome (GRCh38) by UCSC Genome Browser (http://genome.ucsc.edu) [60].

To determine miRNAs derived from TE from human disease and cancer and evolution and primate-related miRNAs, a total of 41 papers were selected from NCBI-PubMed and google scholar with 31 studies on human disease and cancer and 10 studies on evolution and primate-related miRNAs. MiRNAs derived from TE are fully and partially derived from TE, and some of the miRNAs derived from TE share more than one TEs in the sequence.

## 4. Bioinformatic Analyses of Evolution Related Transposable Element-Derived microRNAs

The evolution and primate related miRNAs derived from TE from 10 studies were then localized in UCSC genome browser to check the location in the human genome and which type of TE that miRNAs are derived from (Table 2). From a total of 51 miRNAs derived from TE related with evolution and primates, 16 miRNAs were derived from LINE family, 19 from SINE, 3 from LTR, and 15 miRNAs were derived from DNA transposon. Ten of the miRNAs derived from TE were derived from more than one TEs, and interestingly, hsa-miR-548a-2 and hsa-miR-619 are derived from different types of TEs. For instance, hsa-miR-548a-2 is derived from two LTR16A2 at the terminal of miRNA and one DNA transposon MADE1 is in the middle and hsa-miR-619 has one of each L1MC4 and AluSz6.

From a total of 51 evolution and primate-related miRNAs derived from TE, 21 of miRNAs derived from TE with different type of TEs were chosen for further bioinformatics analysis. The evolution and primate related miRNAs derived from TE were analyzed by ECR browser to briefly check the conservation in chimpanzee, rhesus, mouse, cow, dog, chicken, and zebrafish [72]. Additionally, the structure of each 21 miRNAs derived from TE were predicted by RNAfold webserver which generates the structure of minimum free energy (MFE) contributed by secondary structure of RNA sequences [73]. The strong base-pairing probability shows in color red with value close to 1 and weak base-pairing probability shows in color blue with value close to 0.

**Figure 2:**
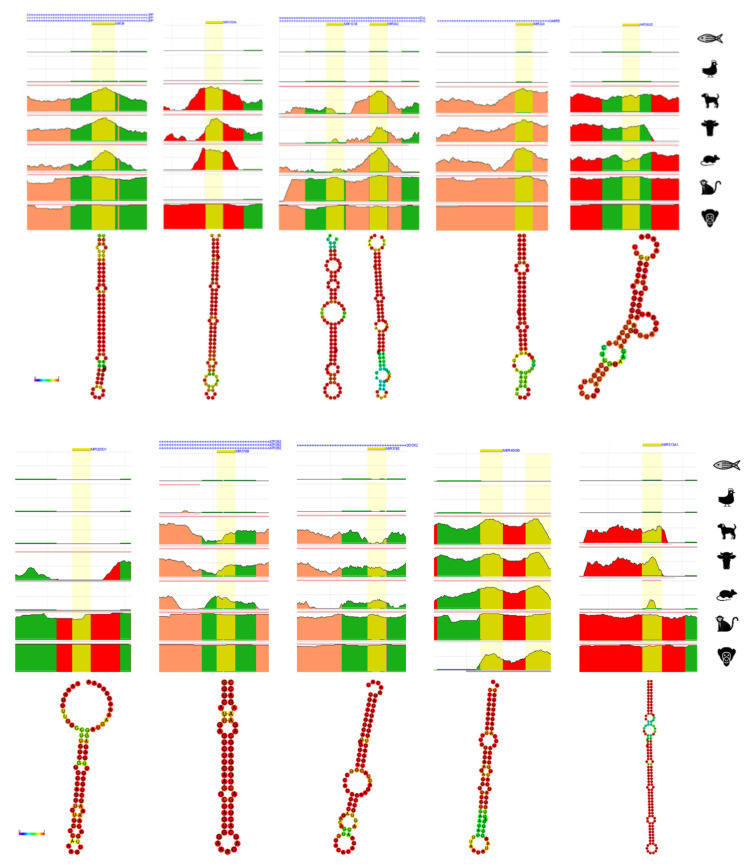

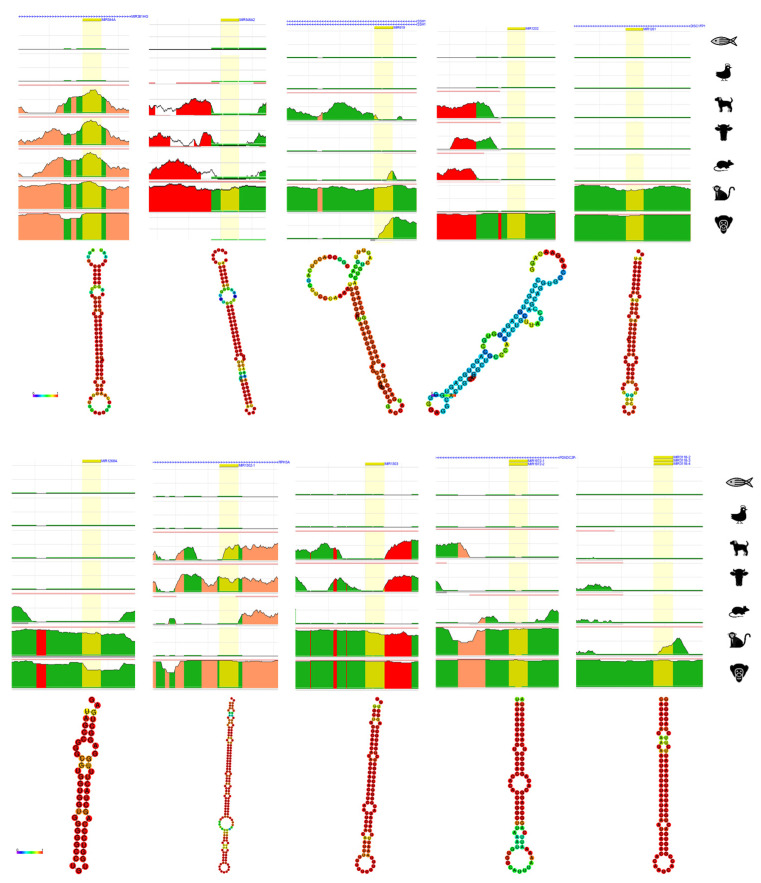
Result of ECR browser and RNAfold on evolution related miRNAs derived from transposable element. On top each miRNA derived from TE, the nearest gene is presented. Yellow represents the region of searched miRNA, green represents transposons and simple repeats, salmon represents intronic region, and red represents intergenic regions. The x-axis represents the position in the human genome and the y-axis represents the conservation scale compared with human. Underneath the ECR browser conservation figure, each RNAhold structure of miRNAs derived from TE is shown. The scale bar on the left shows the minimum free energy (MFE) value of strong and weakest base-pairing values from 0 to 1.

From the numerically ordered list of evolution and primate related miRNAs derived from TE, one of each miRNA was selected from miRNAs derived from the same TE family (hsa-miRNA-28, -130a, -151b, -224, -302e, -320d-1, -342, -378b, -378e, -450b, -513a-1, -544a, -548a-2, -619, -1202, -1261, -1268a, -1302-1, -1303, -1972-1, -3118-3) to examine the conservation throughout human, chimpanzee, rhesus, mouse, cow, dog, chicken and zebrafish (Figure 2). Amongst the 21 of analyzed evolution and primate related miRNAs derived from TE, hsa-miRNA-151b (chr14: 100,109,419-100,109,514) and -342 (chr14: 100,109,655-100,109,753) are located near each other, however, they do not share the same TE family. All 21 of miRNAs derived from TE were not conserved from chicken and zebrafish and hsa-miRNA-28, -224, and -544a was conserved from chimpanzee, rhesus, mouse, cow and dog, hsa-miRNA-342, -302e, -378b, and -378e was conserved in primates but partially conserved from mouse, cow and dog. Hsa-miRNA-1202 and -3118-3 showed conservation only in chimpanzee and hsa-miRNA-548a-2 and -619 showed conservation only in rhesus monkey. Hsa-miRNA-320d-1, -1261, 1268a, -1302-1, -1303 and -1972-1 shows conservation in primates. Strangely, most of miRNAs derived from TE were well conserved in chimpanzee, however, hsa-miRNA-548a-2 was not conserved in chimpanzee, hsa-miRNA-450b and -619 was partially conserved in chimpanzee, and hsa-miRNA-130a, -1202, -1302-1, and -3118-3 was not conserved in rhesus monkey. Hsa-miRNA-513a-1 was not found in mouse genome.

The secondary structure of 21 of evolution and primate related miRNAs derived from TE were predicted by RNAfold webserver [73]. Almost all the MFE structure of miRNAs derived from TE had strong base-pairing MFE values, with the exception of hsa-miRNA-1202 which shows weakest MFE structure.

## 5. Bioinformatic Analyses of Human Diseases Related Transposable Element-Derived microRNAs

The human disease and cancer related miRNAs derived from TE from 31 studies were then localized in the UCSC genome browser to check the location in the human genome and which type of TE that miRNAs are derived from (Table 3). As mentioned previously, miRNAs derived from TE are not only derived from one TE, however, it could be derived from more than one TE with different families. From a total of 34 human diseases and cancer-related miRNAs derived from TE, 16 miRNAs were derived from LINE, 6 from SINE, 2 from LTR, and 10 miRNAs were derived from DNA transposons. Unlike evolution and primate related miRNAs derived from TE which share two types of TEs in one miRNA, no more than one TE type share miRNA from human disease and cancer related miRNAs but 7 out of 34 miRNAs derived from TE share more than one same TE family shares miRNAs derived from TE.

**Figure 3:**
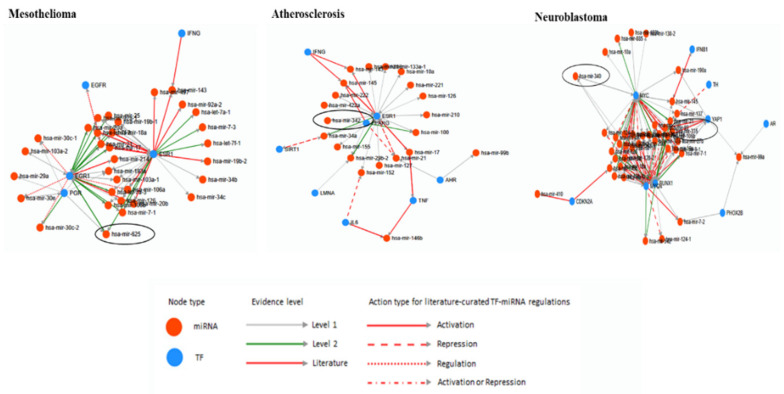
The analyses of human diseases related miRNAs derived from TE and transcription factors by TransmiR. For evidence level, level 1 and 2 is based on ChIP-seq data and level 2 is more stringent than level 1. The Literature level is based on the research papers. Other information is shown in the figure.

The database TransmiR v2.0 was used to predict the correlation of miRNAs and TFs [67]. From all the list of the human disease uploaded in TransmiR database, three diseases Mesothelioma, Atherosclerosis and Neuroblastoma related miRNAs and TF were found (Figure 3). Hsa-miRNA-625 is one of the Mesothelioma-related miRNA and it is activated by EGR1, PGR, and ESR1 TFs. The evidence level of EGR1 and ESR1 is level 2 and it is stricter than level 1 TF PGR. Atherosclerosis related miRNAs derived from TE is hsa-miRNA-342 and it is activated by ESR1 in level of 1. Two miRNAs derived from TE, hsa-miRNA-335 and -340 were related Neuroblastoma. The literature level evidence provided TF MYCN represses hsa-miRNA-335, and YAP1, RUNX1, and MYC activate hsa-miRNA-335 by level of 1. MYCN, RUNX1, and MYC activate hsa-miRNA-340 by level of 1.

**Figure 4:**
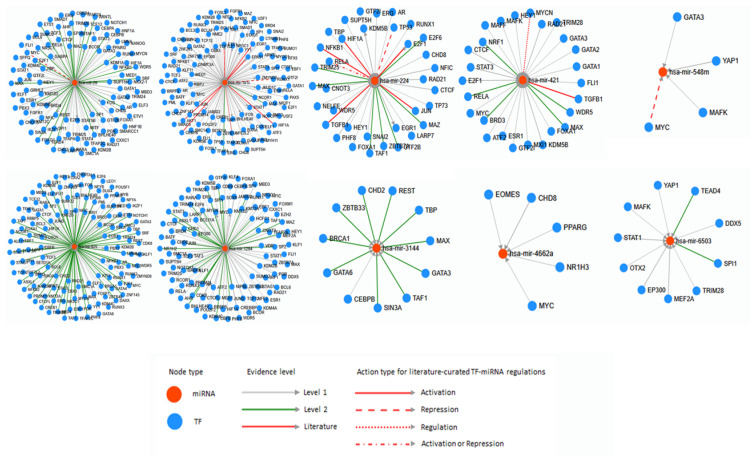
The correlation of miRNAs derived from TE and transcription factors analyzed by TransmiR. For evidence level, level 1 and 2 are based on ChIP-seq data and level 2 is more stringent than level 1. The Literature level is based on the research papers. Other information is shown in the figure.

From the list of human disease and cancer-related miRNAs derived from TE, numerically one of each miRNA was selected (hsa-miRNA-28, -181c, -224, -421, -548m, -625, -1294, -3144, -4662a, and -6503) to analyze by TransmiR v2.0. After all, it also provides correlation between numerous miRNA and TFs (Figure 4). Hsa-miRNA-28 is activated by various TFs, however, MYC represses hsa-miRNA-28 exclusively. Mostly, hsa-miRNA-28 was activated or repressed by TF and similarly, hsa-miRNA-181c was activated by many TFs. On the other hand, hsa-miRNA-181c was also activating abundant TFs. Additionally, RUNX1 and ETS1 represses and NFE2L2 regulates hsa-miRNA-181c. Hsa-miRNA-224 was activated by many TFs and it activates E2F1, EGR1, and KDM5B. TP53 represses and RELA activates or represses hsa-miRNA-224. Hsa-miRNA-421 was activated by most of related TFs, however MYCN regulates hsa-miRNA-421. Hsa-miRNA-548m is activated by GATA3, YAP1, and MAFK however, MYC represses hsa-miRNA-548m. Hsa-miRNA-625, -1294 is activated by abundant TFs and hsa-miRNA-625 activates several TFs. Hsa-miRNA-1294 also activates KMT2D, EP300, and MYC. Hsa-miRNA-3144 is activated by CHD2, SIN3A, GATA6, REST, TAF1, BRCA1, GATA3, MAX, ZBTB33, TBP, and CEBPB and hsa-miRNA-4662a is activated by CHD8, EOMES, MYC, NR1H3, and PPARG. Lastly, MEF2A, TEAD4, OTX2, MAFK, EP300, SPI1, STAT1, TRIM28, YAP1, and DDX5 activates hsa-miRNA-6503.

## 6. Discussion

Bioinformatics tools are useful and important when not much information is provided or studied for the target subjects. Numerous bioinformatics tools are provided online, and are ready to be used right away or downloaded. There are several bioinformatics tools of miRNAs, however, TE related bioinformatics tools are still insufficient. By using bioinformatics database related with TE, the distribution of TE has been modified (Table 1) [15]. The distribution of TE is highly scattered in the genome of most of the species. In the evolutionary aspects on distribution of TE, SVA element is exclusive in humans only. *Alu* element from SINE is a primate and mouse specific element excluding few mammalians (horse, cow, cat, and dog), chicken, zebrafish, and C. elegans. From LINE, the proportion of CR1 element is very low amongst all of mammalians, fish, and C. elegans, however, one study provided the evidence that CR1 element is moderately scattered in avian, crocodilian, turtle, and lepidosaurian, also known as diapsid reptiles [84]. The distribution of ERVK from LTR element presents in primates until rhesus monkey, mouse and cow amongst the mammalians. Most of the ERVK studies are performed in primates, thus mouse shows highest of percentages of ERVK element among all the species, and one study mentioned that human and mouse contain numerous LTR-derived TFBS which contributes in other TFs to bind, and they did not mention the reason why mouse has a high percentage of ERVK element, yet it might be due to embryonic stem cells of mice [85,86,87].

Approximately half of the genome is covered in TEs for mammalians and zebrafish, and over 10 percent for chicken and C. elegans and these highly distributed TEs are capable of generating miRNAs and TFBSs [30,31,34,87]. Based on the miRNA studies, miRNAs derived from TE were filtered into two types, primate and evolution and human disease and cancer. Table 2 shows 51 of primate and evolution related miRNAs derived from TE and Table 3 shows 34 of human disease and cancer-related miRNAs derived from TE. First, to analyze miRNAs derived from TE related in primates and evolution, ECR browser was used to check the conservation on few of selected miRNAs derived from TE. The conservation is influential to miRNAs derived from TE related in primates and evolution due to selecting the target species or samples before going into the actual experiments. Previous studies checked the conservation of each target miRNAs they found to applicate them on primate and evolution related miRNAs [54,70,88]. ECR browsers are used to predict the conservation of the target gene, miRNA, or the specific region of the genome. The conservation on few of the selected primate and evolution-related miRNAs derived from TE show conservation well until mammalians, however, few of miRNAs derived from TE are not randomly conserved (Figure 2). To examine the conservation precisely, the sequence of each miRNA is needed to be downloaded from each species. TargetScan database provides the sequences of conserved miRNAs in the target genes and this method is more accurate than the prediction from ECR browser [89,90]. The RNAfold result predicts the strongness of base pairing as well as the MFE value of the miRNAs by the colors. The miRNA with the weakest structure is predicted as miRNA-1202.

The human disease and cancer related miRNAs derived from TE were analyzed with TFs. TransmiR database provides the information on TFBS that regulates or correlates with miRNAs. First, the examination of all 34 human disease and cancer related miRNAs derived from TE from Table 3 were analyzed to check the correlation between miRNAs derived from TE and TFs by human disease and cancer provided from TransmiR database (Figure 3). Four miRNAs derived from TE were found from three human disease and cancers provided from TransmiR. Other miRNA and TF studies used TransmiR to predict which TFs that their target miRNA is targeting or correlate together and applicate them on further bioinformatics analysis or experiments [91,92]. In addition, few of TFs were determined on human disease and cancer related miRNAs derived from TE. As shown in Figure 4, some miRNAs derived from TE interact with numerous TFs and on the other hand, some miRNAs derived from TE interact with few TFs. The study of miRNA-548m and MYC supported the data of TransmiR based on the result of stroma-inducing miRNA-549m inhibition leads to the c-Myc overexpression [93]. By using TransmiR databases, the hypothesis was suggested that enhancer activity of miRNAs derived from TE is increased by TFs, and the report actually mentioned that the enhancer activity of miRNAs derived from TE OF-miRNA-307 might induced by the TFs near OF-miRNA-307 binds in 3′UTR of target gene [94].

The aim of this study was to introduce the basic bioinformatics tools used for TE and miRNAs derived from TE studies. The evolution and human disease-related miRNAs derived from TE were identified by published papers and they were analyzed with bioinformatics tools. Abundant miRNAs were derived from TEs and they have a close relation with primate and evolution and human disease and cancer. Here, fundamental information of miRNAs derived from TE by using several of the bioinformatics tools are analyzed.

## Figures and Tables

**Table 1:** The distribution of transposable elements in various species.

	Human	Chimpanzee	Gorilla	Orangutan	Gibbon	Macaque	Rhesus	Marmoset	Mouse	Horse	Cow	Cat	Dog	Chicken	Zebrafish	*C. elegans*
Non-TEs	47.5%	49.2%	50.6%	47.9%	48.1%	48.5%	50.7%	51.0%	55.0%	54.8%	50.6%	56.3%	56.8%	88.8%	47.4%	87.4%
SINE/MIR	2.9%	2.9%	3.0%	2.9%	2.9%	2.9%	3.1%	2.7%	0.6%	3.8%	2.3%	3.1%	2.9%	0.0%	0.0%	0.0%
SINE/Alu	10.5%	10.3%	8.8%	10.0%	10.4%	11.0%	10.5%	11.0%	4.8%	0.0%	0.0%	0.0%	0.0%	0.0%	0.0%	0.0%
SINE/other	0.0%	0.0%	0.0%	0.0%	0.0%	0.0%	0.0%	0.0%	2.4%	3.7%	9.4%	8.3%	7.6%	0.0%	2.4%	0.0%
SVA	0.1%	0.0%	0.0%	0.0%	0.0%	0.0%	0.0%	0.0%	0.0%	0.0%	0.0%	0.0%	0.0%	0.0%	0.0%	0.0%
LINE/L2	3.7%	3.8%	3.8%	3.7%	3.8%	3.8%	4.0%	3.3%	0.4%	5.2%	2.7%	4.0%	3.7%	0.0%	1.7%	0.0%
LINE/CR1	0.4%	0.4%	0.4%	0.4%	0.4%	0.4%	0.4%	0.4%	0.0%	0.6%	0.3%	0.4%	0.4%	6.8%	0.0%	0.3%
LINE/RTE	0.2%	0.2%	0.2%	0.2%	0.2%	0.2%	0.2%	0.1%	0.0%	0.3%	12.9%	0.2%	0.2%	0.0%	0.2%	0.0%
LINE/L1	17.5%	17.7%	16.8%	18.3%	17.4%	17.3%	15.5%	18.8%	19.9%	18.0%	13.3%	17.0%	16.8%	0.0%	0.4%	0.0%
LINE/other	0.0%	0.0%	0.0%	0.0%	0.0%	0.0%	0.0%	0.0%	0.0%	0.0%	0.0%	0.0%	0.0%	0.0%	0.5%	0.0%
LTR/ERVK	0.3%	0.3%	0.3%	0.3%	0.2%	0.4%	0.3%	0.0%	4.9%	0.0%	0.5%	0.0%	0.0%	0.0%	0.0%	0.0%
LTR/ERV1	2.9%	2.8%	2.7%	2.9%	2.6%	2.7%	2.8%	2.2%	1.2%	1.9%	1.4%	1.0%	1.0%	0.2%	0.4%	0.0%
LTR/ERVL	5.8%	5.9%	5.8%	5.9%	5.7%	5.8%	6.1%	5.2%	5.9%	5.0%	2.7%	4.0%	3.8%	1.3%	0.0%	0.0%
LTR/Gypsy	0.2%	0.2%	0.2%	0.2%	0.2%	0.2%	0.2%	0.1%	0.0%	0.3%	0.0%	0.2%	0.2%	0.0%	1.7%	0.0%
LTR/other	0.0%	0.0%	0.0%	0.0%	0.0%	0.0%	0.0%	0.0%	0.0%	0.2%	0.0%	0.0%	0.0%	0.0%	3.1%	0.3%
DNA/TcMar	1.5%	1.5%	1.5%	1.5%	1.5%	1.5%	1.6%	1.3%	0.2%	0.8%	0.6%	0.8%	0.7%	0.3%	6.1%	1.8%
DNA/hAT	2.2%	2.2%	2.2%	2.2%	2.2%	2.2%	2.3%	2.0%	0.9%	2.7%	1.6%	2.2%	2.1%	0.5%	9.7%	0.5%
DNA/other	0.0%	0.0%	0.0%	0.0%	0.0%	0.0%	0.0%	0.0%	0.0%	0.0%	0.0%	0.0%	0.0%	0.2%	19.6%	6.1%
Other/Unknown	4.3%	2.6%	3.7%	3.6%	4.4%	3.1%	2.3%	1.9%	3.8%	2.7%	1.7%	2.5%	3.8%	1.9%	6.8%	3.6%
TOTAL: 100%																

**Table 2:** The list of evolution related miRNAs derived from transposable element. The coordinates of miRNAs derived from transposable element (TE) in the human genome, the type and name of TE that miRNAs are derived from, and the references are shown in each column.

	microRNA	Coordinates	Type of TE	References
1	miR-28	chr3:188,688,781-188,688,866	LINE_L2c, L2c	[53,55]
2	miR-130a	chr11:57,641,198-57,641,286	LINE_MamRTE1	[55]
3	miR-151a	chr8:140,732,564-140,732,653	LINE_L2c, L2c	[55]
4	miR-151b	chr14:100,109,419-100,109,514	LINE_L2b	[53,55]
5	miR-224	chrX:151,958,578-151,958,658	DNA_MER135	[53]
6	miR-302e	chr11:7,234,766-7,234,837	SINE_MIR	[70]
7	miR-320d-1	chr13:40,727,816-40,727,887	LINE_L1MEd	[53,55]
8	miR-342	chr14:100,109,655-100,109,753	SINE_MamSINE1	[55]
9	miR-374a	chrX:74,287,286-74,287,357	LINE_L2c	[53]
10	miR-378a	chr5:149,732,825-149,732,890	SINE_MIRc, MIRc	[55]
11	miR-378b	chr3:10,330,229-10,330,285	SINE_MIR3, MIRc	[54,55]
12	miR-378d-1	chr4:5,923,275-5,923,328	SINE_MIRb	[54,55]
13	miR-378d-2	chr8:93,916,022-93,916,119	SINE_MIRc	[54,55]
14	miR-378e	chr5:170,028,488-170,028,566	SINE_MIRb, MIRc	[55]
15	miR-378f	chr1:23,929,070-23,929,147	SINE_MIRc	[54,55]
16	miR-378h	chr5:154,829,458-154,829,540	SINE_MIRc	[55]
17	miR-378i	chr22:41,923,222-41,923,297	SINE_MIRc	[55]
18	miR-450b	chrX:134,540,185-134,540,262	LINE_L1ME4a	[53]
19	miR-466	chr3:31,161,704-31,161,787	LINE_L1ME3	[54]
20	miR-513a-1	chrX:147,213,463-147,213,591	DNA_MER91C	[54]
21	miR-513a-2	chrX:147,225,826-147,225,952	DNA_MER91C	[54]
22	miR-513b	chrX:147,199,044-147,199,127	DNA_MER91C	[54]
23	miR-513c	chrX:147,189,704-147,189,787	DNA_MER91C	[54]
24	miR-518d	chr19:53,734,877-53,734,963	LINE_MamRTE1	[54,71]
25	miR-544a	chr14:101,048,658-101,048,748	DNA_MER5A1	[54]
26	miR-544b	chr3:124,732,439-124,732,516	DNA_MER5A1	[54]
27	miR-548a-1	chr6:18,571,784-18,571,880	DNA_MADE1	[54]
28	miR-548a-2	chr6:135,239,160-135,239,256	LTR_LTR16A2, DNA_MADE1, LTR_LTR16A2	[54]
29	miR-570	chr3:195,699,401-195,699,497	DNA_MADE1	[54]
30	miR-603	chr10:24,275,685-24,275,781	DNA_MADE1	[56]
31	miR-619	chr12:108,836,908-108,837,006	LINE_L1MC4, SINE_AluSz6	[54]
32	miR-637	chr19:3,961,414-3,961,512	LINE_L1MC4a	[57]
33	miR-652	chrX:110,055,329-110,055,426	DNA_MER91C	[54]
34	miR-1202	chr6:155,946,797-155,946,879	LTR_MER52A	[56]
35	miR-1261	chr11:90,869,121-90,869,202	DNA_Tigger1	[54]
36	miR-1268a	chr15:22,225,278-22,225,329	SINE_AluJo	[54]
37	miR-1268b	chr17:80,098,828-80,098,877	SINE_AluSx1	[54]
38	miR-1273c	chr6:154,853,360-154,853,436	SINE_AluJo	[54]
39	miR-1273h	chr16:24,203,116-24,203,231	SINE_AluJb	[54]
40	miR-1302-1	chr12:112,695,034-112,695,176	DNA_MER54	[54]
41	miR-1303	chr5:154,685,776-154,685,861	SINE_AluJr, FLAM_A	[54]
42	miR-1304	chr11:93,733,674-93,733,764	SINE_AluJo	[54]
43	miR-1587	chrX:39,837,561-39,837,613	LTR_MLT1H2	[54]
44	miR-1972-1	chr16:15,010,321-15,010,397	SINE_AluSx, FLAM_A	[54]
45	miR-2355	chr2:207,109,987-207,110,073	LINE_MamRTE1, MamRTE1	[53]
46	miR-3118-1	chr21:13,644,775-13,644,850	LINE_L1PA12	[54]
47	miR-3118-2	chr15:20,832,795-20,832,869	LINE_L1PA14	[54]
48	miR-3118-3	chr15:21,406,385-21,406,459	LINE_L1PA13	[54]
49	miR-3118-4	chr15:21,843,750-21,843,824	LINE_L1PA13	[54]
50	miR-4452	chr4:86,542,482-86,542,552	SINE_AluJo	[54]
51	miR-6303	chr10:24,275,685-24,275,781	DNA_MADE1	[54]

**Table 3:** The list of human disease related miRNAs derived from transposable element. The coordinates of miRNAs derived from TE in the human genome, the type and name of TE that miRNAs are derived from related disorders and the references are shown in each column.

	microRNA	Coordinates	Type of TE	Related Disorders	References
1	miRNA-28	chr3:188,688,781-188,688,866	LINE_L2c, L2c	OC, M	[74]
2	miRNA-95	chr4:8,005,301-8,005,381	LINE_L2b, L2c	OC, BrC	[74]
3	miRNA-130a	chr11:57,641,198-57,641,286	LINE_MamRTE1	LuC, OC, LiC	[46,48,75,76]
4	miRNA-151a	chr8:140,732,564-140,732,653	LINE_L2c	G, OC, BrC, M	[49,74]
5	miRNA-151b	chr14:100,109,419-100,109,514	LINE_L2b	OC, BrC, M	[74]
6	miRNA-181c	chr19:13,874,699-13,874,808	LINE_MamRTE1	LiC, A, OC, BrC	[74,77]
7	miRNA-224	chrX: 151,958,578-151,958,658	DNA_MER135	LiC	[48,51]
8	miRNA-320d-1	chr13:40,727,816-40,727,887	LINE_L1MEd	OC, BrC, M, N	[47,48,74]
9	miRNA-335	chr7:130,496,111-130,496,204	SINE_MIRb	OC, M	[74]
10	miRNA-340	chr5:180,015,303-180,015,397	DNA_MARNA	OC	[74]
11	miRNA-342	chr14:100,109,655-100,109,753	SINE_MamSINE1	EC, ACC, PaC	[48,78]
12	miRNA-361	chrX: 85,903,636-85,903,707	DNA_MER5A	LuC	[52]
13	miRNA-378a	chr5:149,732,825-149,732,890	SINE_MIRc, MIRc	BlC	[49]
14	miRNA-378b	chr3:10,330,229-10,330,285	SINE_MIR3, MIRc	L	[49]
15	miRNA-421	chrX:74,218,377-74,218,461	LINE_L2c, L2c	Ph, Pa	[49]
16	miRNA-513a-1	chrX:147,213,463-147,213,591	DNA_MER91C	LuC	[79]
17	miRNA-513a-2	chrX:147,225,826-147,225,952	DNA_MER91C	LuC	[79]
18	miRNA-518d	chr19:53,734,877-53,734,963	LINE_MamRTE1	LuC	[79]
19	miRNA-545	chrX:74,287,104-74,287,209	LINE_L2c	LuC	[79]
20	miRNA-546b	chr6:119,069,022-119,069,167	DNA_MADE1	LuC	[79]
21	miRNA-548l	chr11:94,466,495-94,466,580	DNA_MADE1	LuC	[79]
22	miRNA-548m	chrX:95,063,141-95,063,226	DNA_MADE1	LuC	[79]
23	miRNA-625	chr14:65,471,102-65,471,186	LINE_L1MCa	M, GC, CoC, LuC, KC	[49,80,81]
24	miRNA-626	chr15:41,691,585-41,691,678	LINE_L1MB8, L1MB8	HPV	[82]
25	miRNA-646	chr20:60,308,474-60,308,567	LTR_LTR67B	LuC	[79]
26	miRNA-659	chr22:37,847,678-37,847,774	DNA_Arthur1B	D	[77]
27	miRNA-1290	chr1:18,897,071-18,897,148	DNA_Tigger4a	LuC	[79]
28	miRNA-1294	chr5:154,347,071-154,347,283	SINE_MIRb	LuC	[79]
29	miRNA-2355	chr2:207,109,987-207,110,073	LINE_MamRTE1, MamRTE1	HPV	[82]
30	miRNA-1304	chr11:93,733,674-93,733,764	SINE_AluJo	LuC, Pr, UCEC	[50,83]
31	miRNA-3144	chr6:120,015,179-120,015,257	LINE_L1MA8	LiC PrC, CeC, HPV	[82,83]
32	miRNA-3681	chr2:12,199,130-12,199,201	LTR_LTR16D1	CeC, LiC	[49]
33	miRNA-4662a	chr8:124,821,985-124,822,051	LINE_L1ME4b	LuC	[49]
34	miRNA-6503	chr11:60,209,071-60,209,156	LINE_MLT1D	LiC, L	[49,83]

Abbreviations: Ovarian cancer (OC), Melanoma (M), Breast cancer (BrC), Lung cancer (LuC), Glioblastoma (G), Alzheimer (A), Neuroblastoma (N), Endocrine cancer (EC), Acrinar cell carcinoma (ACC), Pancreatic cancer (PaC), Bladder cancer (BlC), Leukemia (L), Pheochromocytoma (Ph), Paraganglioma (Pa), Gastric cancer (GC), Colorectal cancer (CoC), Kidney cancer (KC), Human papillomavirus (HPV), Dementia (D), Preeclampsia (Pr), Uterine Corpus Endometrial Carcinoma (UCEC), Prostate cancer (PrC), Cervical cancer (CeC), Liver cancer (LiC).
